# Teething Application

**Published:** 1876-02

**Authors:** 


### TEETHING APPLICATION.

The following is recommended in cases of painful dentition:—

| | | |
|---|---|---|
| ℞. | Syrup of tamarinds, | ʒijss |
| | Infusion of suffron, | ℈ij |
| | Honey | ʒijss |
| | Tinct. (essence) of vanilla, gtt.iv. M. | |

Rub gently over the gums with the finger or rag. An application of a similar character is the following:—

℞. Saffron (powdered), gr.iv-vj

Honey, ʒij-iij.

Glycerine may be substituted for the honey.

